# Clinical and microbiological profile of adult inpatients with community acquired pneumonia in Ilorin, North Central, Nigeria

**DOI:** 10.4314/ahs.v20i4.18

**Published:** 2020-12

**Authors:** Olutobi Babatope Ojuawo, Olufemi Olumuyiwa Desalu, Ademola Emmanuel Fawibe, Ayotade Boluwatife Ojuawo, Adeniyi Olatunji Aladesanmi, Christopher Muyiwa Opeyemi, Mosunmoluwa Obafemi Adio, Abdulraheem Olayemi Jimoh, Dele Ohinoyi Amadu, Abayomi Fadeyi, Kazeem Alakija Salami

**Affiliations:** 1 Department of Medicine, University of Ilorin Teaching Hospital, Ilorin, Nigeria; 2 Department of Paediatrics, University of Ilorin Teaching Hospital, Ilorin, Nigeria; 3 Department of Medical Microbiology, University of Ilorin Teaching Hospital, Ilorin, Nigeria

**Keywords:** Community acquired pneumonia, microbiological profile, Nigeria

## Abstract

**Background:**

The optimal management of community acquired pneumonia (CAP) depends on the clinical and microbiological profile in the locality.

**Objectives:**

To determine the clinical and microbiological profile of patients admitted with CAP in Ilorin, Nigeria.

**Methods:**

One hundred and two consenting consecutively selected patients with clinical and radiologic confirmation of CAP were recruited in 12 months. The socio-demographic, physical examination and laboratory/radiologic parameters were documented in a questionnaire. Microbiological evaluation of their sputum was done and blood samples were taken for complete blood count, culture, serum urea and serological evaluation for atypical bacteria and some viral pathogens.

**Results:**

CAP constituted 5.9% of the total medical admissions during the one-year study period. The mean age of the patients was 49 ± 22 years with the largest frequency in those aged 65 years and above. The commonest symptoms were shortness of breath (96.1%) and cough (94.1%), with a median duration of 3 days from symptom onset to admission. Systemic hypertension was the commonest comorbid illness (25/102; 24.5%). *Klebsiella pneumoniae* was the predominant pathogen isolated (20/102; 28.1%). The susceptible antibiotics were Imipenem, Ceftazidime and Ceftriaxone. Intra-hospital mortality was 17.6%. CURB – 65 score of ≥ 2 and the presence of complications of CAP were the independent predictors of mortality.

**Conclusion:**

CAP constitutes a significant disease burden in Ilorin, Nigeria. Typical bacteria accounted for over half of the pathogens isolated from the patients with gram negative agents predominating. This highlights a possible shift in the microbiological profile which could guide empirical treatment.

## Introduction

Community acquired pneumonia (CAP) is associated with a significant burden of disease globally; accounting for a significant number of hospital admissions and deaths especially in low income.^1^,^2^ The disease is prevalent in Nigeria and is responsible for 2.5% – 5.7% of the medical admissions; as well as between 15.3 and 24.9% of respiratory admission from previous studies in tertiary health facilities in the country.^3^–^6^ The mortality from previous surveys concerning admitted patients with CAP in the country also ranged from 7.4% to 26%.^3^,^4^, ^7^–^9^

Epidemiological reports from sub Saharan Africa also show high levels of morbidity and mortality from the disease as approximately 4 million cases of pneumonia occur annually, resulting in about 200,000 deaths.^10^ Globally, about 450 million people are estimated to be affected with pneumonia every year; accounting for about 7% of the world's deaths.^11^

Despite the availability of antimicrobial agents and effective vaccines, the disease also constitutes the sixth leading cause of death in the United States of America and the number one cause of death from infectious diseases.^2^ The increasing burden of the disease has been linked to the high prevalence of specific risk factors for this infection in patients worldwide. This occurs, mainly, from immunosuppressive illnesses like human immunodeficiency virus (HIV) infection and diabetes mellitus as well as structural lung diseases like chronic obstructive pulmonary disease (COPD), bronchiectasis and cystic fibrosis.^12^

Regarding the microbiological profile, *Streptococcus pneumoniae* was the most frequent isolate in previous studies in the SouthEast and South-South regions of Nigeria.^4^,^13^ However, *Klebsiella pneumoniae* was the commonest pathogen in other studies in the middle belt and South Western regions of the country.^14^,^15^ These surveys however did not evaluate presence of atypical bacterial and viral pathogens.

Generally, the morbidity and mortality associated with CAP is related to the disease severity at presentation, presence of co-morbid illness and delays in starting appropriate drug therapy.^2^ Despite the upsurge of antimicrobial resistance, the mortality associated with the disease can still be significantly mitigated by prompt institution of appropriate therapeutic medications.^2^ Hence, there is a need for information concerning the pattern of presentation, severity, prognostic factors, microbiological profile of pathogens causing CAP in our locality as well as identifying drug susceptibility with the goal of guiding prompt initiation of proper empirical treatment. There is also a paucity of information regarding the common atypical bacterial and viral pathogens causing CAP in our setting despite several previous reports on the disease.

## Materials and methods

### Study Location

This was a hospital based prospective observational study carried out in the emergency unit and adult medical wards of the University of Ilorin Teaching Hospital, Ilorin, Nigeria. The hospital is a 600-bed tertiary healthcare facility in North Central Nigeria and serves as a referral centre for patients in Kwara state as well as other adjourning states.

### Patients

One hundred and two consenting patients with CAP who met the inclusion criteria for the study were recruited consecutively over a one-year period (January - December 2017). The patients recruited were individuals who had new onset of at least two of the respiratory symptoms of cough with or without sputum production, shortness of breath, chest pain and fever. All of them also had evidence of an infiltrate or opacity on plain chest x-ray. Seventy-four patients who had previously been admitted in a healthcare facility in the preceding 14 days prior to onset of current respiratory symptoms were excluded based on the definition of CAP^16^ as well as those who had taken any antibiotic before presentation. Only two patients did not give consent to participate in the study. Recruited patients were followed up from the time of admission to the outcome (discharge or death).

### Data collection and procedures

A structured questionnaire adapted as a modification of the modified Medical Research Council respiratory questionnaire was administered to obtain the patient's demographics and reported symptoms of CAP. A thorough physical examination was also carried out on all recruited subjects. Oxygen saturation at admission was measured on room air using a portable pulse oximeter (New Healthcare Finger Pulse Oximeter, China 2015). The blood pressure (BP) was measured with a mercury sphygmomanometer (Accosson, England) with standard cuff (25cm x 12cm) in supine position on both arms after at least 5 minutes' rest. Two consecutive measurements were obtained about 5 minutes apart and the average was recorded.

A fifteen-milliliter sample of each patient's venous blood was obtained by venipuncture at admission using a vacutainer under aseptic technique prior to commencement of first dose of empirical antibiotic(s). The blood sample was used for complete blood count, culture, serum urea as well as serological evaluation for *Mycoplasma pneumoniae, Chlamydia pneumoniae, Legionella pneumophilia*, Influenza A virus and Respiratory syncytial virus (RSV). Serological evaluation for atypical bacterial and viral pathogens was carried out based on Enzyme Linked Immunosorbent Assay (ELISA) techniques, adhering strictly to the manufacturer's instructions.

Sputum samples were also collected in sterile containers within the first hour of hospital admission in all the individuals who had productive cough. This was done in all recruited patients prior to commencement of the first dose of empirical antibiotics. The subjects were instructed to take deep breaths and cough forcefully from the lungs with expectoration of at least two millilitres of sputum. Collected sputum samples were first inspected macroscopically at the microbiology unit and then under a microscope. This was followed by initial gram staining after which culture was performed if the sputum specimen contained at least 25 neutrophils and less than 10 epithelial cells per high power field. Sputum samples were subsequently liquefied and inoculated into blood, MacConkey and chocolate agar. Thereafter, incubation was done aerobically at 37 degrees Celsius. The blood agar was for isolating *Streptococcus pneumoniae* and *Staphylococcus aureus*; MacConkey agar for Gram negative bacteria like *Klebsiella* species while the chocolate agar was used for isolating fastidious pathogens like *Haemophilus influenzae*. Drug sensitivity testing was done using disc diffusion methods using commonly available antibiotics.

Venous blood (5 millilitres) was inoculated into the brain heart infusion (BHI) broth containing 0.05% sodium polyanetholesulfonate. A minimum of blood to broth ratio of 1:10 was used. This was incubated at 37°C and checked for bacterial growth for up to 7 days. Bottles that showed growth were sub-cultured onto chocolate and MacConkey agar plates. Those with no growth after 7 days were sub-cultured before being labelled negative. The complete blood counts were carried out to obtain the white cell counts using an automated hematology analyzer. The serum urea levels were also assayed. HIV screening was also done after pre-test counselling. The severity of the disease was assessed at admission using the British Thoracic Society (BTS) recommended CURB-65 scoring method.

### Data Analysis

All data obtained were analyzed using a statistical package for social science, IBM SPSS statistics® 2012 version 21.0 for Windows by IBM USA. Demographic and clinical data of cases were summarized using frequencies, percentages and proportions. Normally distributed continuous variables were expressed as means + standard deviations. Chi-square test was used to determine the relationship between the categorical variables. Multivariate binary logistic regression analysis was used to determine the predictors of intrahospital mortality.

### Ethical issues

The study was approved by the Ethical Review Committee of the University of Ilorin Teaching Hospital, Ilorin, Nigeria. Informed consent was obtained from all patients prior to recruitment into the study.

## Results

### Admission breakdown and socio-demographic characteristics of the study population

One hundred and seventy-eight patients were admitted with pneumonia over the study period; 42 of them were excluded due to previous recent hospital admissions. This made the total number of CAP admissions to be 136. The hospital admissions for CAP within the study period constituted 5.9% (136/2297) of the total medical admissions. Thirty-two of the patients with CAP were excluded due to prior antibiotic use following while 2 did not consent to participate in the study.

Eventually, 102 patients were recruited consisting 46 males and 56 females giving a male to female ratio of 1:1.2 (Table 1). The highest frequency in terms of the age group was observed in those aged 65 years and above (34/102; 33.3%). Ninety-five (93.1%) were managed on the medical ward while 7/102 (6.9%) required admission in the Intensive Care Unit (ICU). The months of June, July and August had the largest number of recruited patients with 14, 13 and 16 subjects respectively. (Table 2)

**Table 1:** Socio-demographic characteristics of recruited subjects

Socio-demographic variables	Frequency (%) (n = 102)
**Age group (in years)**	
≤20	12 (11.8)
21–30	15 (14.7)
31–40	17 (16.7)
41–50	8 (7.8)
51–60	12 (11.8)
61–64	5 (4.9)
≥ 65	34 (33.3)
**Mean Age: 49.8 ± 22.1 years**	
**Sex**	
Male	46 (45.1)
Female	56 (54.9)
**Ethnic Group**	
Yoruba	98 (96.1)
Hausa	3 (2.9)
Igbo	1 (1.0)
**Marital Status**	
Single	21 (20.6)
Married	67 (65.7)
Widowed	14 (13.7)

**Table 2:** Monthly breakdown of patients recruited

Month	Frequency (%)
January	5 (4.9)
February	7 (6.9)
March	7 (6.9)
April	8 (7.8)
May	8 (7.8)
June	14 (13.7)
July	13 (12.7)
August	16 (15.7)
September	7 (6.9)
October	7 (6.9)
November	5 (4.9)
December	5 (4.9)
Total	102 (100)

### Comorbid illnesses / risk factors for CAP

Systemic hypertension was the commonest comorbidity with almost a quarter (25; 24.5%) of the subjects affected. This was followed by diabetes mellitus and cigarette smoking in 11(10.8%) each of the patients respectively. Sickle cell disease was also a co-morbid illness/risk factor in 10 (9.8%) of the subjects. Fourteen (13.7%) of them had no co-morbid illness or risk factor for CAP. (Table 3)

**Table 3:** Frequencies of co-morbidities/risk factors for CAP

Co-morbidities/risk factors	Frequency (%)
Systemic Hypertension	25 (24.5)
Diabetes Mellitus	11 (10.8)
Acid Peptic Disease	6 (5.9)
Connective Tissue Diseases	2 (2.0)
Bronchial Asthma	3 (2.9)
Cigarette Smoking	11 (10.8)
Alcohol Ingestion	3 (2.9)
COPD	7 (6.9)
Congestive Cardiac Failure	1 (1.0)
Cancer	3 (2.9)
HIV	6 (5.9)
Sickle cell disease	10 (9.8)
No comorbidity/risk factor	14 (13.7)

### Presenting symptoms of recruited subjects

Shortness of breath was the commonest symptom reported by the patients, occurring in 98 (96.1%) of them. This was followed in a descending order by cough (94.1%), fever (89.2%), sputum production (84.3%) and pleuritic chest pain (41.2%). The median duration from onset of symptoms to hospital admission was 3 days (IQR of 2–7 days).

### Classes of pathogens isolated

There was microbiological confirmation of CAP in 71 (69.6%) patients. Most of the pathogens isolated were typical bacterial pathogens alone (33; 32.3%). Viral agents and atypical bacterial pathogens alone were isolated in 11.8% and 10.7% of the subjects respectively. Mixed or double pathogens were detected in 13.7% of the patients.

### Pathogens and mode of isolation

Twenty-one (20.6%) pathogens were isolated through sputum culture alone, 11 (10.8%) pathogens were isolated through blood culture alone while 2 (2.0%) pathogens were isolated from both sputum and blood culture (Table 4). Overall, *Klebsiella pneumoniae* (14.7%) was the most common single isolate followed by RSV (11.8%). *Legionella pneumophilia* was the most common single atypical bacteria isolated (7.8%).

**Table 4:** Pathogens and mode of isolation

Organisms and mode of isolation	Frequency (%)
***Sputum Culture alone (n=21; 20.6%)***	
*Klebsiella pneumoniae*	8 (7.8)
*Streptococcus pneumoniae*	6 (5.9)
*Staphylococcus aureus*	2 (2.0)
*Pseudomonas aeruginosa*	2 (2.0)
*Klebsiella oxytoca*	2 (2.0)
*Candida albicans*	1 (1.0)
***Blood Culture alone (n=11; 10.8%)***	
*Klebsiella pneumoniae*	6 (5.9)
*Staphylococcus aureus*	4 (3.9)
*Pseudomonas aeruginosa*	1 (1.0)
***Sputum and Blood Culture (n=2; 2%)***	
*Klebsiella pneumoniae*	1 (1.0)
*Staphylococcus aureus*	1 (1.0)
***Serology alone (n=24; 23.5%)***	
*Legionella pneumophilia*	8 (7.8)
*Mycoplasma pneumoniae*	3 (2.9)
*RSV*	12 (11.8)
*Legionella pneumophilia and RSV* (double pathogens from serology)	1 (1.0)
**Mixed/Double pathogens (Sputum and or blood culture + Serology)**	
***Typical bacteria isolated from blood culture in combination with*** ***atypical bacteria/viruses isolated from serology (n=3; 2.9%)***	
*Staphylococcus aureus and Legionella pneumophilia*	1 (1.0)
*Staphylococcus aureus and RSV*	2 (2.0)
***Typical bacteria isolated from sputum culture in combination with*** ***atypical bacteria/viruses isolated from serology (n=7; 6.9%)***	
*Klebsiella pneumoniae and Legionella pneumophilia*	2 (2.0)
*Klebsiella pneumoniae and RSV*	1 (1.0)
*Klebsiella oxytoca and Legionella pneumophilia*	1 (1.0)
*Staphylococcus aureus and Influenza A virus*	1 (1.0)
*Streptococcus pneumoniae and Influenza A virus*	1 (1.0)
*Streptococcus pneumoniae and RSV*	1 (1.0)
***Typical bacteria isolated from both sputum and blood culture in*** ***combination with atypical bacteria/viruses from serology (n=3; 2.9%)***	
*Klebsiella pneumoniae and Chlamydia pneumoniae*	1 (1.0)
*Klebsiella pneumoniae and Legionella pneumophilia*	1 (1.0)
*Staphylococcus aureus and Legionella pneumophilia*	1 (1.0)

### Complications and markers of severity of CAP

Table 5 shows that in all, 67 complications of CAP were identified across 63 (61.8%) of the patients with the commonest being hypoxaemia (50.7%; 34/67). There was radiographic evidence of multilobe lung involvement and the presence of bacteremia in 29.4% and 15.7% of the patients respectively. The median WBC count was 9.35 x 109/L (IQR 6.78 – 15.20) and almost half (45.1%) demonstrated leukocytosis on complete blood count.

**Table 5:** Complications of CAP in recruited subjects

Complication	Frequency (%)
Hypoxaemia	34 (50.7)
Parapneumonic effusion	9 (13.4)
Empyema thoracis	4 (5.9)
Sepsis	16 (23.9)
Lung Abscess	2 (3.0)
Massive haemoptysis	2 (3.0)
Total	67 (100)

• Some patients had more than one complication

### Drug sensitivity pattern

Sixteen (80%) of the 20 isolates of Klebsiella pneumoniae were sensitive to Imipenem while 13 (65%) each were sensitive to Ceftazidime and Ceftriaxone respectively (Table 6). However, the gram-positive pathogens isolated (*Staphylococcus aureus* and *Streptococcus pneumoniae*) were most sensitive to Amoxicillin/Clavulanic acid.

**Table 6:** Drug sensitivity pattern

	Cipro	Ceftria	AmC	Gent	Ceftaz	Imi	Pip/Taz	Azith	Cefurox
***Klebsiella*** ***Pneumoniae*** ***(20)***	10 (50)	13 (65)	10 (50)	12 (60)	13 (65)	16 (80)	NT	10 (20)	10 (10)
***Staphylococcus*** ***aureus (12)***	5 (41.7)	9 (75)	10 (83.3)	9 (75)	5 (41.7)	NT	NT	8 (66.7)	9 (75)
***Streptococcus*** Pneumoniae (8)	5 (62.5)	3 (37.5)	7 (87.5)	4 (50)	4 (50)	NT	NT	6 (75)	6 (75)
***Klebsiella*** ***oxytoca (3)***	2 (66.7)	1 (33.3)	1 (33.3)	3 (100)	3 (100)	3 (100)	NT	1 (33.3)	1 (33.3)
***Pseudomonas*** ***aeruginosa (3)***	2 (66.7)	0	0	1 (33.3)	3 (100)	3 (100)	3 (100)	1 (33.3)	0

**Cipro** – Ciprofloxacin, **Ceftria** – Ceftriaxone, **AmC** – Amoxicillin/Clavulanic acid, **Gent** – Gentamicin, **Ceftaz** – Ceftazidime, **Imi** – Imipenem, **Pip/Taz** – Piperacillin Tazobactam, **Azi** – Azithromycin, **CefuroxCefurox** – Cefuroxime, **NT** – Not tested.

### Predictors of mortality during admission period

About three-quarters of the patients were discharged while the percentage mortality was 17.6% (95% CI: 9.49 -25.78). Table 7 shows that the mean age of the patients who died during the admission period was significantly higher than those who survived the illness (60.65 ± 21.90 vs. 47.60 ± 21.66 years; p=0.026). Also, the proportion of patients with CURB-65 scores of greater than or equal to 2 was significantly higher in those who succumbed to the illness when compared to those that survived (p=<0.001). The percentage of patients with hypoxemia was also significantly higher in patients who died when compared with those who survived (p=<0.001).

**Table 7:** Relationship between clinical outcome of patients and their socio-demographic/clinical parameters

Parameter	Patients alive during admission period (%) N =85	Mortalities during admission period (%) N=17	Total N =102	Test statistics	p-value
**Mean Age (years)**	47.60 ± 21.66	60.65 ± 21.90		0.640^a^	**0.026**

**Sex**					
Male	35 (41.2)	11 (64.7)	46 (45.1)	3.168^b^	0.075
Female	50 (58.2)	6 (35.3)	56 (54.9)		

**Body Mass Index**					
Underweight	8 (9.4)	2 (11.8)	10 (9.8)	1.882^c^	0.597
Normal	56 (65.9)	8 (47.1)	64 (62.7)		
Overweight	19 (22.4)	1 (5.9)	20 (19.6)		
Mild Obesity	1 (1.1)	0 (0)	1 (0.9)		

**CURB-65 score**					
0–1	53 (5.9)	2 (0)	55 (4.9)	14.59^c^	**<0.001**
≥2	32 (56.5)	15 (23.5)	47 (51.0)		

**Site of patient** **treatment**					
Wards	81 (95.3)	14 (82.3)	95 (93.1)	3.712^d^	0.089
ICU	4 (4.7)	3 (17.6)	7 (6.9)		

**Presence of** **comorbidities**					
Yes	32 (37.6)	4 (23.5)	36 (35.3)	1.236^b^	0.266
No	53 (62.4)	13 (76.5)	66 (64.7)		

**SpO2 (%)**					
≤ 90	21 (15.3)	13 (76.5)	34 (33.3)	17.082	**<0.001**
>90	64 (75.3)	4 (3.9)	68 (66.7)		

a independent student t-test

b chi-square

c yates corrected chi-square

d fisher's exact test

Likewise, as shown in Table 8, the proportion of patients with involvement of 2 or more lung lobes was also significantly greater in those that died when compared to patients that survived (p=0.020). The multivariate binary logistic regression analysis illustrated in Table 9 shows that CURB-65 score of ≥2 and the presence of complications of CAP were the identified independent predictors of mortality (p = 0.020 and 0.004 respectively).

**Table 8:** Relationship between clinical outcome of the patients and their laboratory/radiologic parameters

Parameter	Patients alive during admission period (%) N=85	Mortalities during admission period (%) N = 17	Total N =102	Test statistics	p-value
**Presence of** **complications**					
Yes	63 (74.1)	0 (0)	63 (61.8)	32.954^b^	**<0.001**
No	22 (25.9)	17 (100)	39 (38.2)		
**Number of lung** **lobes affected**					
1	64 (75.2)	8 (47.1)	72 (70.6)	5.440^c^	**<0.020**
≥2	21 (24.7)	9 (52.9)	30 (29.4)		
**Presence of** **bacteraemia**					
Yes	12 (14.1)	4 (23.5)	16 (15.7)	0.949^b^	0.330
No	73 (85.9)	13 (76.5)	86 (84.3)		
**WBC count** **(×10^9^/L)**					
< 4 and >30	8 (9.4)	1 (5.9)	9 (8.8)	0.219^b^	0.639
4–30	77 (90.6)	16 (94.1)	93 (91.1)		
**Isolation of** **microbe**					
Yes	60 (70.6)	11 (64.7)	71 (69.6)	0.232^b^	0.630
No	25 (29.4)	6 (35.3)	31 (30.4)		
**Group of microbes**					
Typical bacteria alone	28 (32.9)	5 (29.4)	33 (32.4)	2.263^c^	0.943
Typical and atypical bacteria	5 (5.9)	2 (11.8)	7 (6.9)		
Typical bacteria and viruses	5 (5.9)	1 (5.9)	6 (5.9)		
Atypical bacteria only	10 (11.4)	1 (5.9)	11 (10.8)		
Atypical bacteria and Viruses	1 (1.2)	0 (0)	1 (0.9)		
Viruses only	11 (12.9)	1 (5.9)	12 (11.8)		
Fungi	0 (0)	1 (5.9)	1 (0.9)		
None	25 (29.4)	6 (35.3)	31 (30.4)		

b chi-square

c yates corrected chi-square

**Table 9:** Multivariate logistic regression to determine the predictors of intra hospital mortality

Variables	B	p-value	OR	95% CI
Age of patient	0.015	0.486	1.015	0.973 – 1.059
CURB-65 score – 2	2.672	**0.020**	14.472	1.523 – 137.562
Involvement of 2 or more lung lobes	0.170	0.814	1.185	0.286 – 4.909
Presence of complications of CAP	3.254	**0.004**	25.885	2.788 – 240.337
Presence of hypoxaemia	0.867	0.239	2.381	0.562 – 10.092
Constant	-1.707	0.271	0.181	

OR – odds ratio

95% CI – 95% confidence interval

## Discussion

This study highlights the substantial burden of CAP in Ilorin, Nigeria, accounting for 5.9% of medical admissions in the hospital and occurring mostly in the elderly population. There was microbial detection in over two-thirds of our patients with gram - negative typical bacterial pathogens especially *Klebsiella pneumoniae* predominating from the isolates.

The preponderance of CAP in individuals older than 65 years supports findings from earlier observations that the disease is more common in elderly people.^3^,^7^,^17^,^18^ This can be explained by the higher risk of micro aspirations in elderly patients during sleep from depressed cough and glottis reflexes, the less efficient airway mucociliary clearance, the presence of comorbidities which serve as risk factors for CAP as well as the waning immunity in this unique population. There was also a slight female predominance which is in accordance with the report by Mbata et al^7^ that assessed the severity of CAP in the South Eastern region of Nigeria. The frequent and high intensity rainfall periods of May to August experienced the highest number of CAP cases which is in synchrony with a previously established relationship between cooler air temperature and increased CAP cases in the southern region of the country.^19^

Furthermore, over three quarters of our patients had co-existing illnesses majority of which also doubled as risk factors for CAP. This included systemic hypertension, diabetes mellitus, cigarette smoking and sickle cell disease. The high number of co-morbid diseases surpassed related findings in previous retrospective studies^3^,^9^ on CAP in the country. The prospective nature of this study which helped circumvent issues relating to missing data may explain the higher number of comorbidities recorded.

Regarding the presenting symptoms of the patients, shortness of breath and cough dominated as the principal symptoms. This was in accordance with previous investigations on CAP by Le Bel et al^20^ and Muller et al^21^ but contrary to the report by Onyedum et al^3^ where fever and cough constituted the cardinal presenting symptoms of CAP. The challenge of recollecting the presence and sequence of symptoms, the varying cultural perception of the illness by the patients as well as the severity of the illness at presentation may have also contributed to the inconsistency of reported symptoms. Also, the subjective and potentially limiting nature of dyspnoea may have contributed to it being the most common presentation in our study.

We also observed that about half of our patients were admitted in hospital despite having a CURB - 65 score of 0 or 1. This was largely due to a number of patients presenting with other markers of severity of CAP such as hypoxaemia and multilobe lung involvement. A significant number of patients also had background medical co-morbid illnesses which contributed to hospital admission. The patients with CURB-65 score of 4 were managed in the ICU while 3 of the initial 10 patients with CURB-65 score of 3 had clinical deterioration while on treatment on the medical ward culminating in ICU admissions. The fact that a large percentage of our patients admitted for CAP had at least one complication was not particularly surprising considering the tertiary healthcare setting of this study.

Concerning the microbiological findings, the overall yield of aetiologic pathogens of CAP (71; 69.6%) was higher than figures reported from previous studies in Nigeria 4,14,15,22 and this relatively higher yield of pathogens could be due to the fact that multiple microbiological assays including sputum, blood and serological assessment for atypical bacteria and viruses were included in this present report. The prompt collection of sputum and blood culture samples before the first dose of empirical antibiotics may also have resulted in an increased microbial yield.

*Klebsiella pneumoniae* was the commonest single isolate in this study; corroborating previous reports in the South Western and middle belt regions of Nigeria.^14^, ^15^,^22^, ^23^ This was however contrary to some other reports^4^,^13^,^24^ where *Streptococcus pneumoniae* was the main isolate. The dominance of *Klebsiella pneumoniae* in this study buttresses the need for further investigation into a possible change in the epidemiological trend of aetiologic agents of CAP in this environment. Additionally, gram negative organisms are recognized to cause severe pneumonia in elderly patients 25, 26 and are also more common in patients with co-morbid illnesses.^27^ Hence, the fact that a third of our patients (33.3%) were over 65 years of age and 86.3% of the patients had comorbidities could have contributed to our discovery. Though *Haemophilus influenzae* is a recognized cause of CAP worldwide^28^ it was not isolated in this survey as has equally been the experience in previous studies in the country.^3^,^4^,^29^ The fastidious nature of the pathogen in sputum culture may have been responsible for its non-isolation.

Viruses were isolated in 18.6% of the subjects, either solely or in combination with another bacterial pathogen with RSV being the predominant viral pathogen detected. This figure was consistent with previous reports by Mandell et al^30^ and Musher et al^31^. It was however lower than figures observed by Lieberman et al^32^ and Gadsby et al^33^ who had larger number of patients (183 and 323 patients respectively) and utilized more sophisticated molecular techniques for isolation of pathogens such as PCR. Also, the fact that the viral serologic assay in this survey was limited to RSV and Influenza A virus may account for the reduced number of viral agents detected compared to the aforementioned studies in which a broader range of viruses were sought.

Mixed or double pathogens were found in 13.7% of subjects and this was consistent with observations from a review article in Europe by Torres et al^34^ who reported frequencies of CAP with mixed etiology to vary between 0.4 – 19.9% across countries in Europe. The finding is also in line with the observation by De Roux et al^35^ who reported that mixed infections occur in greater than 10% of patients admitted with CAP.

Overall, the finding of *Legionella pneumophilia* and RSV as the leading atypical bacterium and virus respectively in this study provides a strong template for further research regarding atypical and viral CAP in Nigeria and the West African sub-region; which up till now is largely restricted to the developed world. This study also provides a guide as to the choice of empirical agent to be employed prior to getting culture results. Individuals demonstrating aforementioned risk factors for gram negative pathogens may also benefit from use of third generation cephalosporins instead of the widely used penicillins and macrolides. However, it is clear from our observations that penicillins are still advocated in cases of hospitalized patients with gram-positive CAP.

About three quarters of the recruited patients were discharged while the percentage mortality was 17.6%. The percentage mortality of admitted patients was more than that of previous reports from South East Nigeria3 and East Africa^36^ which reported mortality rates of 11.9% and 11% respectively. This may be due to the relatively significant proportion of elderly patients in this study. The percentage mortality in this study is however less than the 26% reported by Tanimowo et al^9^ from patients managed in Osogbo, South Western Nigeria. This may possibly be due to the fewer number of patients with CAP (65) reviewed in their study.

CURB - 65 scores ≥ 2 and the presence of complications of CAP were the identified independent predictors of mortality. This is not surprising as the validated CURB-65 score is a recognized marker of disease severity and predictor of mortality.^37^ The presence of complications is also known to worsen disease severity and confer worse prognosis.^38^

Regarding limitations of our study, the interpretation of the results of may be affected by the fact that it was conducted in a single centre with 102 patients. Also, we did not capture antibiotic history outside that related to the index illness which may also have affected the spectrum of microbes isolated. However, this study still significantly contributes to the body of knowledge for many reasons. Firstly, ours is a prospective study unlike the previous studies which were mainly retrospective data analyses of patients treated earlier and many of them involved even fewer patients. Furthermore, most of them were published more than 5 years ago and considering the fact they were retrospective analysis of patients treated earlier than the time of publication, they may not adequately reflect the current trends in adult patients with CAP. Another crucial reason in support of this work is the fact that, to the best of our knowledge, it is the first study in Nigeria and especially in the North Central region to test for atypical bacteria and viral pathogens in the aetiology of CAP among our adult population. Finally, our findings will serve as a template for further multicenter studies in the future; all of which will assist with formulating guidelines for treatment of CAP in our setting and in Africa as a whole.

## Conclusion

CAP remains a significant cause of morbidity and mortality with admissions more common among the elderly population. Typical bacterial pathogens constitute the commonest cause of CAP in our setting with gram-negative pathogens, especially *Klebsiella pneumoniae*, predominating. While the carbapenems and third generation cephalosporins demonstrate good antibiotic susceptibility for the gram-negative pathogens, amoxicillin/clavulanic acid and cefuroxime still demonstrate good sensitivity for the gram-positive pathogens. These germane observations will serve as a guide to clinicians in our setting with regards to the empirical treatment of admitted patients with CAP.
